# Syringe Service Program Utilization, Behavioral, and Experiential Factors Associated with Greater Naloxone Protection in a Longitudinal Cohort of People Who Use Illicit Opioids in New York City

**DOI:** 10.21203/rs.3.rs-7926376/v1

**Published:** 2025-10-31

**Authors:** Alex S. Bennett, Yuyu Chen, Adrian Harris, David Frank, Saba Rouhani, Alice Cepeda, Alverado Valdez, Jemar Bather, Melody Goodman, Luther Elliott

**Affiliations:** New York University; New York University; New York University; New York University; New York University; New York University; New York University; New York University; New York University; New York University

**Keywords:** overdose, naloxone protection, drug supply, syringe service program, harm reduction, drug test strips

## Abstract

**Background:**

Despite efforts to blanket high-mortality communities with no-cost naloxone, drug checking technologies, and alternatives to using opioids alone, there remains a considerable lack of knowledge about how exposure to these low-threshold interventions may impact rates of naloxone protection.

**Methods:**

People who used illicit opioids in New York City were surveyed between April 2019–April 2022. *Naloxone protection* was defined as the proportion of opioid use events in the past 30 days when opioid use occurred with naloxone present and someone to administer it. Mixed-effect Poisson models examined correlates (syringe service program utilization, behavioral, and experiential factors) of naloxone protection among the overall sample and stratified by gender.

**Results:**

Among 428 study participants (mean age 49 years, 64% cisgender male, 35% Black, 41% Latinx), several factors were significantly associated with naloxone protection levels. Visiting a syringe service program (SSP) in the last three months was significantly associated with higher naloxone protection in the full sample (IRR = 1.94, 95% CI: 1.38–2.74) and among males (IRR = 1.84, 95% CI: 1.29–2.64). Significantly lower naloxone protection was also observed among Latinx participants compared to White individuals in the full sample (IRR = 0.64, 95% CI: 0.44–0.94) and among females aged 57–72 years compared to those aged 20–40 years (IRR = 0.39, 95% CI: 0.16–0.95). Participants living with someone who does not use opioids (vs. living with someone who uses opioids) showed significantly lower naloxone protection levels in the full sample (IRR = 0.54, 95% CI: 0.37–0.80) and among males (IRR = 0.46, 95% CI: 0.29–0.73). Other factors associated with lower naloxone protection in the full sample included any opioid withdrawal in the last 30 days, higher pain severity, and more negative life events. Conversely, receiving social support from another person who uses opioids was significantly associated with higher naloxone protection in the full sample (IRR = 1.14, 95% CI: 1.07–1.22) and among females (IRR = 1.30 95% CI 1.16–1.46). Additional factors associated with greater naloxone protection in the full sample included: concurrent opioid and stimulant use, heroin/injection use (1–15 days), fentanyl test strip use, lifetime opioid overdose, and number of lifetime arrests.

**Conclusion:**

Findings suggest positive impacts of syringe service program engagement and use of harm reduction best-practices and interventions on promoting naloxone protection and highlight differential associations. These results can be used to inform culturally-sensitive and tailored outreach to people at risk of overdose.

## Introduction

With close to 100,000 overdose deaths nationwide reported each year in 2021–2024, communities across the United States (U.S.) continue to experience alarming levels of overdose fatalities [1]. While overdose mortality rates have leveled or declined in some regions, fatalities among socially disadvantaged and racially minoritized populations remain at historic highs in many areas, highlighting ongoing inequities [2–5]. Moreover, while men in general have higher rates of substance use and overdose mortality than women, rates of overdose among women have increased by 480% between 1999 and 2021 [6] and women experience more health consequences and lower rates of service utilization [7–9]. The response to the overdose crisis has been piecemeal [10, 11], and although there has been an acceleration in the deployment of low-threshold harm reduction services in response to the overdose crisis over the past five years [12], questions remain about the extent of uptake of these services and how uptake relates to people’s opioid-related decision making and other behaviors.

A central lifesaving feature of the national response to the overdose crisis has been the provision of no-cost naloxone to people at risk for overdose, as well as their friends and family [13]. Since the 1990s, syringe service programs (SSPs) have been the principal vectors of overdose prevention education and naloxone distribution while also helping to facilitate social relationships among people who use drugs. As a medication that was first distributed to community members in the 1990s, especially to people who use drugs, naloxone has become more accessible in the past decade [14, 15]. In the U.S., 55% of SSPs had implemented overdose education and naloxone distribution (OEND) programs by 2013; by 2019, 94% had implemented OEND [10]. Despite considerable obstacles, including federal bans on funding and not-in-my-back-yard advocacy (NIMBYISM), early SSP overdose prevention programs established distribution models, focusing on getting naloxone to people who use opioids in community settings where they lived and interacted [14, 16]. SSPs also facilitate social relationships across racial/ethnic, gender, geographic boundaries and adapt to the changing drug supply and needs of people who use drugs [17]. As overdose evolved as a serious public health concern, many SSPs began to offer fentanyl and xylazine test strips, drug checking, safe smoking supplies, and in several communities, safe spaces for monitored substance use [18, 19].

Despite the established lifesaving benefits of naloxone and increased availability through public health departments, SSPs, and increasingly, via vending machines [20], public wall-mounted communal boxes [21], and mail [22], research shows that rates of uptake and possession of naloxone among people who use illicit opioids remains low among Black and Latinx populations, especially women [9, 23]. A recent NYC study, for example, found that relative to Whites, Latinx and Black participants had significantly lower rates of naloxone training, possession, and daily access. Moreover, relative to White individuals, Black and Latinx participants, in particular, women, had significantly lower rates of “naloxone protection” – opioid use events with both naloxone and someone to administer it present [9]. Research has identified barriers to naloxone carriage and uptake including stigma [24], insecurity about competence to use it [25], fear of police involvement, and concerns about unnecessarily precipitating withdrawal [26, 27], especially as higher dose naloxone products (e.g., 8mg and 10mg) are being introduced. NIMBYISM and redlining may also limit uptake and acceptance of naloxone [28–30],, which may be further reinforced by policing practices [31, 32]. Unsurprisingly given these findings, the rate of Black and Latinx overdose mortality in NYC exceeds that of Whites [33].

The observed disparity in naloxone uptake is compounded by the social nature of an overdose reversal itself: not only is naloxone needed to reverse an overdose, but a critical lifesaving feature involved in naloxone rescue is the presence of someone else with knowledge of how to use it, something absent when one is consuming drugs alone [34]. According to a recent NYC data brief, roughly 59% of overdoses in 2023 occurred in a private home, a context in which where bystander intervention is not possible [33]. Thus, even with widespread naloxone access, people who live alone and those who are socially isolated may be less likely to be able to have both naloxone and someone to administer it, the gold standard of “naloxone protection [13, 34].” For women, solitary use can reflect additional layers of gendered stigma, trauma histories, and safety concerns—many women report using alone to avoid violence, exploitation, or judgment from partners and peers [35]. These same factors may account for low service utilization rates among women. A recent study of a Miami SSP found that only 26% of participants were women and women faced elevated injection and sexual health risks [7].

While a growing body of research documents naloxone awareness, ownership, and naloxone carriage, contextual dimensions such as the extent to which a trusted bystander is also available to administer the naloxone in the case of an overdose, is less frequently captured [36, 37]. There also remain open questions about how to harmonize instrumentation used to measure naloxone coverage (e.g., own it, have it, carry it) and how exposure or uptake translates to protection [38, 39]. Study designs to capture changes in naloxone protection over time across and within sociodemographic subcategories have been lacking and thus limit our understanding of how to address disparities. To realize the full potential of naloxone saturation in the overdose crisis, reasons for gaps in naloxone access and gaps in naloxone protection must be better understood and addressed.

This study seeks to shed additional light on barriers and facilitators of naloxone protection, drawing on the study team’s operationalization of naloxone protection as a measure of the past 30 days of opioid use events that were protected by both naloxone and someone to administer it [40]. In doing so, we examine how SSP exposure and utilization of low-threshold harm reduction strategies and best practices may be related to naloxone protection while also looking at the extent to which observable gender, racial/ethnic, socioeconomic, and experiential barriers are associated with naloxone protection over time.

## Methods

### Study Design and Setting

Participants were 18 years or older who reported past 72-hour use of illicit opioids and resided within the five boroughs of NYC and were recruited using respondent-driven sampling for a two-year longitudinal study examining naloxone uptake and utilization [40, 41]. Study participants were enrolled during 2019–2020 and given coupons enabling them to recommend up to three people in their opioid-using network. Potential study participants were screened, and opioid use was verified using a rapid urinalysis Multi-Drug One Step Cup II from BTNX.com, which included nine fentanyl-class drugs in addition to heroin/morphine, benzodiazepines, alcohol, amphetamines, oxycodone, cannabis/tetrahydrocannabinol, and methadone metabolites. Eligible participants provided informed consent and enrolled in the study at a field site at a central Manhattan location, where they completed the baseline survey administered by a trained interviewer who entered responses on a computer. Participants completed the baseline and the first of 24 surveys at enrollment and were compensated $60 cash for the baseline and their first $20 credit to a Clinical Trial Payer card, which was used for subsequent follow-up assessment completions. Additional information about the study methods can be found elsewhere [34, 40, 42, 43]. Participants for this analysis include those who completed the baseline assessments (baseline and first monthly) and at least 2 other monthly assessments over the 24 months of study participation.

### Measures

#### Dependent Variable

##### Proportion of Opioid Use Events Protected by Naloxone.

At baseline and in monthly follow-ups, study participants were asked the number of days in the past 30 days that they used illicit opioids and the average number of times they used each day.

Interviewers multiplied these quantity and frequency values to estimate the monthly total number of illicit use events, verified this estimate with participants, and then asked how many of those total events involved “both naloxone and someone else you trust to administer it was present.” Dividing this number of protected events by total use events created a “proportion of protected events” [40].

#### Independent Variables

##### Demographic and Drug Use Variables.

The survey asked participants about their age in years which were recoded into baseline quartiles (20–40, 41–50, 51–56, 57–72). Race/ethnicity was categorized as non-Hispanic White, non-Hispanic Black, Hispanic/Latinx [Latinx], or non-Hispanic Other (Asian, Native Hawaiian, multiracial). Additional demographic variables included gender, educational attainment (did not complete high school, high school diploma/GED, some college or higher), length of illicit opioid use (measured continuously in years), overdose history (lifetime and past 30 day), and current living situation (alone, with non-using other(s), with other(s) who uses heroin or other opioids).

##### Substance Use and Withdrawal.

Study measurement included baseline and follow-up assessments of substances used in the past 30 days as well as questions about route of administration, polysubstance use, medications for opioid use disorder, and opioid injection drug behaviors [42, 44]. Opioid-related withdrawal was assessed with a single 0–30 survey item, indicating days during which participants experienced at least some symptoms of withdrawal and recoded as none, moderate (1–7 days), and severe (>7 days in the past 30 days). Participants could note all the drugs they took in the past 30 days, as well as polysubstance use/concurrent use of two, three, four, of five or more drug combinations, with and without alcohol.

##### Lifetime Overdose Experiences.

At baseline, participants were asked about lifetime overdose experiences, on any drug and opioid involved. When reading the question to participants, opioid involved overdose was defined for participants: “By ‘overdose’ we mean that a person lost consciousness, stopped breathing or was unresponsive as a result of taking any kind of opioid (e.g. heroin, any prescription opioid pills, methadone or Suboxone), with or without any other drugs or alcohol.”

##### SSP Utilization.

The frequency with which participants visited a SSP in the past three months at baseline was coded as “never ” or “yes,” which included “less than once a month” and “almost daily/daily.”

##### Network Size.

Opioid-using network members were defined using the inclusion criteria for the study. We measured baseline network size using two indicators: 1) the participant-reported number of adults (>18 years of age) whose names they knew (and who also knew their names) that live in NYC and were known to have used illicit opioids in the last three days, and 2) whom the participant had seen at least once in the past two weeks. Because the recruitment of participants was network-based, all participants had at least one network member (the person who had referred them to the study by providing an RDS coupon), and we considered opioid-using network size (≥ 5: “yes” or “no”).

##### Social Support.

Because the size of social networks may be an imprecise indicator of how intimate or supportive these networks are, we included items about the frequency of reported forms of social and material support they had received from their non-drug-using (e.g., “your relatives, non-drug using friends and/or neighbors, program staff”) and drug-using network members in the past three months, including offers of a place to sleep, gifts of money with no strings attached, and emotional support when they were unhappy. We created separate dichotomous past 30-day indicators for each. To investigate how the constitution of networks may relate to overdose risk and protection, we also made a combined categorical support indicator defined as having received none of the forms of support from using and non-using networks; receiving ≥1 form of support from drug-using but no support from non-using network; receiving ≥1 form of support from non-using but no support from drug-using network; and receiving ≥1 form of support from both networks. Finally, participants reported if they were currently cohabiting with a romantic partner, cohabiting with anyone, and if that cohabiting person used opioids.

##### Fentanyl Test Strip.

Participants reported the number of days in the past 30 in which they used fentanyl test strips to check their drugs during the number (N) of opioid use events.

##### Past Adverse Events.

Negative life events represent a subset of the most severe negative experiences drawn from the most recent revision of the Life Events Inventory [45] and included the death or serious illness of your spouse or partner; the death or serious illness of a close friend or family member; a recent altercation with law enforcement and/or arrest; a new conviction; a new period of unemployment or loss of a job; a new debt; a loss of housing; a new period of going unsheltered; a divorce; and a family break-up. The past-month occurrence of 1 or more of the above was coded as yes in the analyses.

##### Lifetime Arrests and Incarceration.

Participants self-reported the number of arrests in lifetime, categorized into low (0–2 times), medium (3–9 times), and high tertiles (10+ times). Lifetime incarceration was the self-reported number of days in jail/prison in lifetime.

##### Severity of Pain.

Pain Severity was assessed using the Brief Pain Inventory [46] and scored according to the instrument’s user guide [47]. Four items with 0–10 response scales corresponding to the severity of pain: 1) currently experienced, 2) at its worst, 3) least, and 4) average levels over the past week. A composite variable calculated as the mean of those four items (range 0–10) was used in the analysis, aligning with earlier operationalizations of the metric [48].

#### Analytic Strategy

We computed descriptive statistics on all variables, using counts and percentages for categorical variables and means and standard deviations for continuous measures. In order to estimate individual trajectories, increase stability and reliability of findings, and reduce noise and measurement error, consistent with previous analyses, we included only participants who had completed three or more assessments (i.e., baseline and two or more follow-ups) in the analytic sample [48]. We implemented inverse probability weighting to address loss to follow-up and minimize selection bias [49]. We constructed a binary variable such that ‘1’ indicated if the participant had three or more study assessments and ‘0’ if the participant had fewer than three study assessments. We regressed this binary outcome on the following baseline characteristics: utilizing an SSP in the last three months, race, gender, age, education, having an opioid-using network size ≥5, social support score (non-user and user), current living situation, length of opioid use, lifetime opioid overdose, and adverse childhood event score. This model was fit among the entire cohort and used to generate inverse probability weights. We incorporated these weights into all analyses of the analytic sample.

Our final selection of independent variables for the multivariable model was both data- and theory-driven. We found important relationships between certain variables at baseline, many of which were theoretically relevant, building on significant baseline findings [40]. We excluded current homelessness and lifetime incarceration from the multivariable models due to collinearity with living situation and lifetime arrest. We also removed several mental health measures as they warrant a separate analysis.

We modeled the outcome (proportion of protected events) as a rate assuming a Poisson distribution, which is more appropriate for non-negative variables than the normal distribution [50]. We conducted bivariate associations between each independent variable’s baseline measure and naloxone protection using mixed-effects Poisson models with participant-level random effects and inverse probability weights. This same statistical method was used to evaluate the longitudinal relationships of SSP utilization, behavioral, and experiential factors with naloxone protection. Since the protectiveness outcome is a proportional outcome (range 0–100), that is derived as the count of naloxone protection out of a total number of opioid exposures in the past 30 days, a log of the 30-day opioid use exposure was used as an offset term with a log link and incorporated in the mixed-effects Poisson regression model. This was done to accommodate the change in the denominator (numbers of days of opioid use in the past 30 days) across participants. We performed gender-stratified analyses to assess differential associations with naloxone protection between males and females in the sample, as the literature shows sex differences in the utilization of SSPs and other harm reduction resources [7, 8]. We explored race stratification, but we did not include a race stratified model because the interaction term between race and the primary exposure of interest was not significant in the full model. We conducted sensitivity analyses of models without inverse probability weights. We estimated incidence rate ratios (IRR) with 95% confidence intervals (CIs). Statistical significance was assessed as P<0.05. Statistical analyses were performed in R version 4.4.2 (R Core Team, R Foundation for Statistical Computing).

## Results

### Baseline Characteristics

The study sample included 428 participants (Table 1), with a mean age of 49.4 years (SD = 13.1). The majority were male (64.2%), Black (35%) or Latinx (41%), and had at least a high school diploma (76.2%). A high portion (76.2%) reported having an opioid-using network of five or more individuals. Living arrangements included 25.1% who lived with another person using illicit opioids; 34.4% lived with someone who did not use opioids, 24.4% lived alone, and 16.2% reported no stable arrangements/currently homeless. Naloxone protection at baseline was low among the overall sample, with 19.2% of past 30-day opioid use events protected by naloxone, though females (23.1%) reported slightly more protection than males, 17%. The mean days of using heroin alone in the past 30 days is 15.3 days (SD = 11.7). Despite low levels of naloxone protection, close to 40% of participants reported using a SSP in the past 3 months. Overall, the sample reported use of overdose prevention strategies when using opioids in the past 30 days: fentanyl test strip use (mean = 7.3 days, SD = 10.9 days), use of test shots (mean = 2.1 days, SD = 5.6 days), and using drugs in public or semi-public places so someone can find you if you overdose (mean = 1.2 days, SD = 4.5 days). The sample had extensive drug use experience with a mean length of illicit opioid use 24.9 years (SD = 12.7 years). Slightly over half of participants (52.5%) experienced moderate or severe opioid withdrawal in the past 30 days at baseline and 36% had experienced a lifetime opioid overdose. Almost one-half of the sample (46.3%) reported experiencing negative life events in the past 30 days at baseline. The majority of the sample reported lifetime arrests with 36% reporting between 3–9 arrests and 31% reporting 10 or more arrests over the lifetime. Similarly, 31% of participants reported having been incarcerated for more than 365 days across their lifetimes.

### Unadjusted Associations with Naloxone Protection at Baseline

Unadjusted analyses (Table 2) indicated that *higher naloxone* protection at baseline in the full sample was associated with SSP utilization in the last three months (IRR = 2.63, 95% CI: 2.51, 2.76), female gender identity (IRR = 2.06, 95% CI: 1.97, 2.15), older age (e.g., 41–50 IRR = 1.98, 95% CI: 1.87, 2.10), higher education (HS diploma/GED IRR = 1.15, 95% CI: 1.08, 1.22; some college education or greater IRR = 1.53, 95% CI: 1.44, 1.62), concurrent use of opioids with cocaine/amphetamines (past 1–15 days IRR = 1.84, 95% CI: 1.73, 1.96; 16–30 days IRR = 1.41, 95% CI: 1.31, 1.51), social support from a user (IRR = 1.01, 95% CI: 1.00, 1.02), living situation (lives with another opioid user IRR=1.79, 95% CI: 1.68, 1.90), lifetime opioid overdose (IRR = 2.00, 95% CI: 1.91, 2.09), and lifetime arrest (high IRR = 1.07, 95% CI: 1.01, 1.13). Conversely, *lower naloxone protection* at baseline correlated with race/ethnicity (Black IRR = 0.54, 95% CI: 0.51, 0.58; Latinx IRR = 0.78, 95% CI: 0.73, 0.82; Other IRR = 0.32, 95% CI: 0.29, 0.36), higher non-user social support (IRR = 0.90, 95% CI: 0.89, 0.91), current living situation (living with no-user IRR = 0.57, 95% CI: 0.54, 0.62; homeless IRR = 0.67, 95% CI: 0.62, 0.73), opioid withdrawal (moderate IRR = 0.43, 95% CI: 0.41, 0.45; severe IRR = 0.60, 95% CI: 0.56, 0.64), and lifetime arrest (medium IRR = 0.72, 95% CI: 0.68, 0.77).

### Longitudinal Associations with Naloxone Protection

In the full sample (Table 3), *higher naloxone protection* was significantly associated with SSP utilization in the last three months (IRR = 1.94, 95% CI: 1.38, 2.74), receiving social support from another person who uses opioids (IRR = 1.14, 95% CI: 1.07, 1.22), concurrent use of any opioids with cocaine/amphetamine (1–15 days IRR = 1.11, 95% CI: 1.08, 1.15; 16–30 days IRR = 1.32, 95% CI: 1.28, 1.37), heroin injection (1–15 days IRR = 1.60, 95% CI: 1.49, 1.72), use of fentanyl test strips (IRR = 1.02, 95% CI: 1.02, 1.02), lifetime overdose (IRR = 1.58, 95% CI: 1.17, 2.13), and lifetime arrest (medium IRR = 1.90, 95% CI: 1.08, 2.18). By contrast, in the full sample, *lower naloxone protection* was significantly associated with race/ethnicity (Latinx IRR = 0.64, 95% CI: 0.44, 0.94), social support (non-user IRR = 0.89, 95% CI: 0.83, 0.95), living situation (living with a non-user IRR = 0.54, 95% CI: 0.37, 0.80), heroin injection (16–30 days IRR = 0.92, 95% CI: 0.86, 0.98), opioid withdrawal (moderate IRR = 0.80, 95% CI: 0.78, 0.82; severe IRR = 0.67, 95% CI: 0.64, 0.71), pain severity score (IRR = 0.96, 95% CI: 0.96, 0.96), and negative life events (IRR = 0.77, 95% CI: 0.76, 79).

In the stratified model among males (Table 3), *higher naloxone protection* was associated with SSP utilization (IRR = 1.84, 95% CI: 1.29, 2.64), concurrent use of opioids and cocaine/amphetamine (1–15 days IRR = 1.22, 95% CI: 1.17, 1.27; 16–30 IRR = 1.22, 95% CI: 1.16, 1.28), heroin injection (1–15 days IRR = 2.60, 95% CI: 2.33, 2.89), use of fentanyl test strips (IRR = 1.03, 95% CI: 1.02, 03), lifetime overdose (IRR = 1.71, 95% CI: 1.19, 2.48), and lifetime arrest (medium IRR = 2.28, 95% CI: 1.45, 3.60; high, IRR = 2.04, 95% CI: 1.29, 3.23). Conversely, *lower naloxone protection* among males was associated with higher education (high school graduate IRR = 0.46, 95% CI: 0.28, 0.73; some college IRR = 0.47, 95% CI: 0.29, 0.73), living situation (lives with a non-user IRR = 0.46, 95% CI: 0.29, 0.73), opioid withdrawal (moderate IRR = 0.77, 95% CI: 0.74, 0.79; severe IRR = 0.78, 95% CI: 0.72, 0.84), pain severity (IRR = 0.96, 95% CI: 0.95, 0.96), and negative life events (IRR = 0.86, 95% CI: 0.83, 0.88).

Among females, *higher naloxone protection* was associated with receiving social support from another user (IRR = 1.30, 95% CI: 1.16, 1.46), concurrent use of opioids and cocaine/amphetamine (16–30 days IRR = 1.36, 95% CI: 1.30, 1.42), and use of a fentanyl test strip (IRR = 1.01, 95% CI: 1.01, 1.01). Conversely, *lower naloxone protection* among females was associated with age (57 to 72 IRR = 0.17, 95% CI: 0.05, 0.55; 51–56 IRR=.39, 95% CI: 0.16, 0.95), social support from a non-user (IRR = 0.75, 95% CI: 0.66, 0.84), heroin injection (1–15 days IRR = 0.89, 95% CI: 0.80, 0.98), moderate and severe opioid withdrawal (moderate IRR = 0.94, 95% CI: 0.90, 0.98; severe IRR = 0.63, 95% CI: 0.59, 0.68), pain severity (IRR = 0.96, 95% CI: 0.95, 0.97) and negative life events (IRR = 0.71, 95% CI: 0.68, 0.74).

The direction of associations was generally consistent between males and females in the stratified models (Table 3) with several notable exceptions. Unlike in the full sample and in males, SSP utilization among females was not significantly associated with lower naloxone protection (IRR = 0.72, 95% CI: 0.42, 1.23). For males, more education was significantly associated with lower naloxone protection (high school graduate or GED IRR = 0.46, 95% CI: 0.28, 0.73; some college or greater IRR = 0.47, 95% CI: 0.29, 0.76), while for females, more education was associated with considerably higher naloxone protection (some college or greater IRR = 2.31, 95% CI: 1.22, 4.36). Among the overall sample and for males, older age was associated with higher naloxone protection, though the relationship was not statistically significant. For women, older age was significantly associated with lower protection (IRR = 0.17, 95% CI: 0.05, 0.55).

Results in models (full sample and stratified by gender) run without inverse probability weighting were similar to weighted models and did not change significant findings (Supplementary Tables 1 and 2).

## Discussion

In this study, we sought to understand predictors of naloxone protection – opioid use events that were protected by naloxone and someone to administer it in the case of an overdose – among a cohort of people who used illicit opioids in NYC. There were positive findings highlighting the potential benefits of SSP utilization on other harm reduction behaviors like naloxone protection in our full sample and among males. There were also discernible disparities in protection observed in the full sample showing that Latinx participants had significantly lower rates of naloxone protection than their White counterparts, a finding consistent with prior research [51–53]. Moreover, our stratified model revealed additional gender disparities with women 51–56 and 57–72 significantly less protected by naloxone than males. Communities have grappled with the best ways to curb the crisis of overdose mortality, since well before the 2017 CDC declaration that the overdose crisis was a public health emergency, debating the relative merits and potential harms associated with various treatment and carceral interventions, and the role of harm reduction services [14, 16]. The present study’s finding, consistent with other recent work [54, 55], that SSP utilization is associated with greater naloxone protection provides strong support for greater investment into these public health sites while also underscoring the need to address observed gender disparities in utilization.

Though males and females reported roughly equivalent baseline SSP use in our sample, our variance in findings between males and females longitudinally with lower rates of protection among females suggests the importance of more investment into understanding gender disparities in harm reduction utilization. A body of research suggests that there is less uptake of available harm reduction resources, including sexual and reproductive healthcare, among females than males who access SSPs [8, 56]. Our findings that older age females, especially those 57–72, have significantly lower naloxone protection, is a strong indication that investment effort needs to be made to reach females in their communities with overdose prevention education and naloxone. At the same time, this finding demonstrates the need for policy and programmatic innovation to support females who use drugs at brick at mortar harm reduction agencies and highlights the need to create settings that are more conducive to engaging women [57, 58]. This may involve a range of strategies including having more female peers and clinicians as well as creating more gender-separated space where sexual and other forms of trauma are less likely to occur [57, 59, 60]. Taken together, these findings reinforce the importance of having a wide range of harm reduction interventions available in many different settings. Findings also point to the need for the implementation of policies that support universal access to a mix of low threshold harm reduction services especially in community settings beyond SSPs to mitigate drug-related harms [61, 62].

Overall and in our stratified models, participants who reported past 30-day use of opioids and cocaine/amphetamines at the same time were significantly more likely to be protected by naloxone than those who reported no days. This is a notable finding and speaks to the success of various outreach efforts in educating people who use drugs on the potential harms of polysubstance use and dangers associated with the false sense of safety observed by some who mistakenly believe stimulants or amphetamines can counter the sedative effects of opioids. This was also the case for people who reported injecting heroin 1–15 days, which was associated with greater naloxone protection. Interestingly, however, there was significantly lower naloxone protection for people who reported 16–30 days of heroin injection. While clearly more research is needed to disentangle these opposite directions observed in levels of protection and frequency of use, it may be that those people who inject drugs more regularly either feel they are less susceptible to overdose (due to the regularity of their use, tolerance) or that being protected is simply not feasible for those who regularly inject due to living arrangements, housing insecurity and other structural factors. Overall and for women, reports of days using heroin alone were associated with less protection, perhaps an indication by some for a preference for solitary use as has been found in other studies [63].

We found that the use of a fentanyl test strip was positively associated with being protected by naloxone, suggesting a clustering of harm reduction practices to minimize risk of overdose mortality. While there are close to 20 SSPs in the NYC area, by December 2023, there were over 300 Opioid Overdose Prevention Programs (OOPP) across NYC offering no-cost naloxone [64] and many also distribute drug test strips. Furthermore, in NYC outreach teams frequently canvas places where people who use drugs congregate in order to distribute harm reduction resources including test strips [65]. Our findings that test strip use is associated with greater naloxone protection suggest that participants considered fentanyl test strips as complementary to naloxone protection, part of an overdose prevention toolkit to draw on in the context of an unregulated and increasingly synthetic drug supply. This is consistent with the findings of other studies that have found positive associations between drug checking programs and harm reduction behaviors to prevent opioid overdose including using less, using with others, or ensuring naloxone availability, especially after a positive fentanyl test [66–69].

In addition to the gender disparities noted, our finding that Latinx overall are significantly less likely to be protected than Whites and Blacks raises important questions about the relative penetration of harm reduction into some communities and barriers some groups face. A recent study found that some NYC neighborhoods have lower than average naloxone distribution rates among non-Latino Black and Latino residents, especially in Queens where the harm reduction infrastructure is less robust than areas of Manhattan and the Bronx [70]. Our findings about the limited rates of protection among the Latinx population and the lack of harm reduction access in some neighborhoods suggests new forms of outreach need to be implemented to address ongoing structural barriers, especially as service delivery faces increased challenges reaching minoritized populations. The literature has identified an expanded need especially in some NYC communities for culturally appropriate interventions, ones that are responsive to language and other barriers many ethnic groups face [71, 72].

Our finding that lifetime arrest was associated with higher levels of protection, rather than less, may be an example of a policy success in the model of naloxone distribution achieved in NYC where no-cost naloxone is available across settings and points of service contact including criminal justice and reentry venues. For example, the New York Police Department officers began carrying naloxone as early as 2014–2015 and by 2018, NYC Health and Hospital reported over 13,000 naloxone kits had been dispensed through jail visitor centers[73], increasing the likelihood that participants with carceral exposures might also have access to naloxone. Early Overdose Prevention trainings also emphasized the heightened risks for overdose after a period of incarceration; a message widely disseminated to people who were incarcerated or those using re-entry services. This message may have been internalized by many, resulting in a greater propensity to use opioids in protected contexts. Taken together, the association between arrest and higher levels of naloxone protection may come from participants knowledge and cumulative experiences with carceral exposures, cycling in and out of jail, wanting to protect themselves from death. Clearly more research is needed to unpack these findings about the associations between forms of carceral exposure and naloxone protection and how low threshold naloxone access across institutional and community-based settings may increase the ability for people to protect themselves.

Higher levels of protection were observed among those who received support from another person who uses drugs. This finding stands in sharp contrast to our findings that receiving support from, or living with, a non-drug user is associated with less protection. This contrast may be an indication that stigma and the fear of being outed as a person who uses drugs to someone who does not use drugs weighs considerably on decisions about selecting contexts of use and whether or not someone asks another person who does not use drugs to “spot” them during a use event. The pervasive shame and stigma that continues to be associated with drug use may be driving people who use drugs outside of the home or underground into even riskier contexts for use [74, 75]. This finding also indicates a needed expansion of safe places and strategies for monitored or protected drug use, including OPC and mobile/telehealth overdose prevention resources. Taken together, these findings are consistent with the literature highlighting the critical role of people who use drugs as key first responders in overdose situations [34].

Negative life events, pain, and withdrawal are also associated with decreased naloxone protection in our study. The lower protection observed in the context of pain, withdrawal, and negative life events is especially concerning considering research also indicates that these factors disproportionately impact socially disadvantage communities and are also associated with increased overdose risk [48]. An exception to this in our model is having experienced a past lifetime overdose, which was associated with greater protection across our sample, suggesting a more nuanced understanding of proximal and distal overdose risks and potentially delayed temporal impacts of the overdose experience is needed [76, 77].

Collectively, study findings and gaps in protection identified point to the critical importance of making available a range of harm reduction resources and approaches for people to select based on known drug supply risks, availability of, and access to, harm reduction resources, individual needs, contexts, and known constraints [using alone]. Agencies in NYC like the Overdose Prevention Center, OnPoint, as well as numerous SSPs and outreach teams are now providing a harm reduction buffet of low threshold options to prevent and/or respond to overdose in a range of community venues. However, they remain underutilized and for many, inaccessible resources to help mitigate the overdose mortality crisis due to a range of barriers including ongoing stigma and structural factors [54, 78]. As part of a comprehensive opioid safety and harm reduction strategy, emphasizing the community health aspect of services obtained at an SSP may help mitigate the historical stigma associated with “syringes” and the dominant framing of people who use drugs as “junkies.” This reframing may help to draw more people who use drugs into low threshold health care rather than reinforcing their status as outsiders in communities in which they live [74].

While the focus of this study was to measure the associations between hypothesized demographic, behavioral, experiential, and substance use practices and our outcome, naloxone protection, the study did not capture the extent to which unprotected use was associated with participants’ desire to use alone, due to safety concerns and/or due to mistrust of others, as noted in other research [35]. More research is needed to better understand motivations for solitary drug use and how services like mobile overdose response might address this need. Another limitation of this study is that data are self-reported and subject to recall bias and limited to people who use unregulated illicit opioids in NYC, limiting generalization. Because follow-up data was collected via phone or other electronic devices, those people who struggled to keep their phones may be underrepresented in the final analyses. The study recruitment also stopped short of the target due to COVID mandated closures in March 2020, limiting the ability of persons who lost phones to meet in-person with study staff on a regular basis. Despite these limitations, the study has many strengths, including the use of novel measures including naloxone protection, and the longitudinal nature of the study that allowed us to capture change over time.

## Conclusions

Due to the recent and rapid introduction of a range of harm reduction drug services into public health infrastructures, and ongoing gender and racial disparities in overdose mortality and overdose prevention service utilization, a better understanding of the barriers and facilitators people who use drugs face in securing contexts for protected opioid use is needed. Without this, harm reduction intervention implementation efforts may not maximize reach and effectiveness. For naloxone to be able to fulfill its promise, wider access and uptake are clearly needed, and institutions and social networks of people who use drugs need to be harnessed such that more opioid use events can be truly protected.

## Supplementary Material

Supplementary Files

This is a list of supplementary files associated with this preprint. Click to download.

- SupplementaryTable1.docx

## Figures and Tables

**Table 1:** Baseline characteristics of 428 participants who had three or more study assessments.

Characteristic	N	N = 428	Male, N = 272 (64.2%)	Female, N = 152 (35.8%)	p-value^1^
**Protection with naloxone Percentage, Mean (SD)**	422	19.2 (35.2)	16.7 (32.8)	23.1 (38.6)	0.089
**Visiting a SEP in the Last 3 Months, No. (%)**	427				0.91
Never		260 (60.9%)	167 (61.6%)	92 (60.5%)	
Yes or Other Categories		167 (39.1%)	104 (38.4%)	60 (39.5%)	
**Race, No. (%)**	423				0.12
White		82 (19.4%)	43 (16.1%)	37 (24.3%)	
Black		148 (35.0%)	93 (34.8%)	55 (36.2%)	
Latinx		173 (40.9%)	116 (43.4%)	55 (36.2%)	
Other		20 (4.7%)	15 (5.6%)	5 (3.3%)	
**Gender, No. (%)**	424				
Male		272 (64.2%)			
Female		152 (35.8%)			
**Age, No. (%)**	428				0.22
20 to 40		117 (27.3%)	67 (24.6%)	47 (30.9%)	
41 to 50		102 (23.8%)	73 (26.8%)	28 (18.4%)	
51 to 56		103 (24.1%)	65 (23.9%)	38 (25.0%)	
57 to 72		106 (24.8%)	67 (24.6%)	39 (25.7%)	
**Education, No. (%)**	428				0.23
Did Not Complete High School		102 (23.8%)	58 (21.3%)	43 (28.3%)	
High School Graduate or GED Program		175 (40.9%)	112 (41.2%)	61 (40.1%)	
Some College or Greater		151 (35.3%)	102 (37.5%)	48 (31.6%)	
**Has Opioid-Using Network Size ≥5, No. (%)**	428	326 (76.2%)	207 (76.1%)	115 (75.7%)	0.99
**Social Support Score (Non User), Mean (SD)**	428	4.2 (2.3)	4.4 (2.4)	3.9 (2.2)	0.048
**Social Support Score (User), Mean (SD)**	428	3.6 (2.4)	3.7 (2.4)	3.4 (2.3)	0.14
**Living Situation, No. (%)**	427				<0.001
Live Alone		104 (24.4%)	70 (25.7%)	31 (20.5%)	
Lives with non-using other(s)		147 (34.4%)	102 (37.5%)	44 (29.1%)	
Lives with other (s) who uses heroin or other opioids		107 (25.1%)	49 (18.0%)	58 (38.4%)	
No stable arrangements / homeless		69 (16.2%)	51 (18.8%)	18 (11.9%)	
**Use any opioids AND a cocaine or amphetamine type stimulant at the same time in the past 30 days, No. (%)**	252				0.45
0 days		147 (58.3%)	92 (55.4%)	54 (63.5%)	
1–15 days		69 (27.4%)	48 (28.9%)	21 (24.7%)	
16–30 days		36 (14.3%)	26 (15.7%)	10 (11.8%)	
**Inject heroin in the past 30 days, No. (%)**	418				0.80
0 days		262 (62.7%)	169 (63.5%)	92 (62.2%)	
1–15 days		51 (12.2%)	30 (11.3%)	20 (13.5%)	
16–30 days		105 (25.1%)	67 (25.2%)	36 (24.3%)	
**Use a fentanyl test strip, Mean (SD)**	427	7.3 (10.9)	6.6 (10.2)	8.3 (11.9)	0.14
**Length of Opioid Use, Mean (SD)**	427	24.9 (12.7)	25.6 (12.5)	24.0 (12.9)	0.20
**Opioid Withdraw in the last 30 days, No. (%)**	427				0.88
None		203 (47.5%)	128 (47.2%)	75 (49.3%)	
Moderate		175 (41.0%)	111 (41.0%)	61 (40.1%)	
Severe		49 (11.5%)	32 (11.8%)	16 (10.5%)	
**Pain Severity Score, Mean (SD)**	428	4.3 (3.4)	4.2 (3.4)	4.6 (3.3)	0.22
**Lifetime Overdose, No. (%)**	428	165 (38.6%)	101 (37.1%)	61 (40.1%)	0.61
**Lifetime Opioid Overdose, No. (%)**	428	154 (36.0%)	97 (35.7%)	54 (35.5%)	0.99
**Negative Life events, No. (%)**	428	198 (46.3%)	129 (47.4%)	66 (43.4%)	0.49
**Lifetime Arrest, No. (%)**	428				<0.001
Low		145 (33.9%)	72 (26.5%)	71 (46.7%)	
Medium		152 (35.5%)	99 (36.4%)	51 (33.6%)	
High		131 (30.6%)	101 (37.1%)	30 (19.7%)	
**Lifetime Incarceration, No. (%)**	428				<0.001
0 days		84 (19.6%)	41 (15.1%)	42 (27.6%)	
1–365 days		213 (49.8%)	120 (44.1%)	90 (59.2%)	
More than a year		131 (30.6%)	111 (40.8%)	20 (13.2%)	
**Days Using Heroin AJone, Mean (SD)**	428	15.3 (11.7)	16.7 (11.4)	13.0 (11.9)	0.002

1 Welch Two Sample t-test; Pearson’s Chi-squared test

**Table 2:** Bivariate associations between naloxone protection and baseline characteristics among the 428 participants who had three or more study assessments, overall and by gender.

	Full Sample (N=422)		Male (N=−268)		Female (N=150)	
Characteristic	Unadjusted IRR (95% CI)^1^	p-value	Unadjusted IRR (95% CI)^1^	p-value	Unadjusted IRR (95% CI)^1^	p-value
**Visiting a SSP in the Last 3 Months**						
Never	–		–		–	
Yes or Other Categories	2.63(2.51, 2.76)	**<0.001**	2.9(2.72, 3.10)	**<0.001**	2.34(2.19, 2.51)	**<0.001**
**Race**						
White	–		–		–	
Black	0.54(0.51, 0.58)	**<0.001**	0.44(0.41, 0.48)	**<0.001**	0.84(0.76, 0.93)	**<0.001**
Latinx	0.78(0.73, 0.82)	**<0.001**	0.61(0.57, 0.66)	**<0.001**	1.2(1.10, 1.30)	**<0.001**
Other	0.32(0.29, 0.36)	**<0.001**	0.17(0.14, 0.20)	**<0.001**	1.38(1.18, 1.60)	**<0.001**
**Gender**						
Male	–		–		–	
Female	2.06(1.97,2.15)	**<0.001**				
**Age**						
20 to 40	–		–		–	
41 to 50	1.98(1.87, 2.10)	**<0.001**	2.51(2.31, 2.73)	**<0.001**	1.91(1.75, 2.08)	**<0.001**
51 to 56	1.73(1.63, 1.85)	**<0.001**	2.31(2.11, 2.53)	**<0.001**	1.25(1.14, 1.37)	**<0.001**
57 to 72	1.29(1.20, 1.37)	**<0.001**	1.86(1.70, 2.04)	**<0.001**	0.95(0.85, 1.05)	0.33
**Education**						
Did Not Complete High School	–		–		–	
High School Graduate or GED	1.15(1.08, 1.22)	**<0.001**	1.02(0.94, 1.12)	0.62	1.68(1.54, 1.83)	**<0.001**
Some College or Greater	1.53(1.43, 1.62)	**<0.001**	1.62(1.49, 1.77)	**<0.001**	1.63(1.48, 1.78)	**<0.001**
**Has Opioid-Using Network Size ≥5**						
No	–		–		–	
Yes	1.03(0.97, 1.09)	0.31	0.91(0.85, 0.98)	**0.009**	1.26(1.16, 1.37)	**<0.001**
**Social Support Score (Non User)**	0.9(0.89, 0.91)	**<0.001**	0.96(0.95, 0.97)	**<0.001**	0.83(0.81, 0.84)	**<0.001**
**Social Support Score (User)**	1.01(1.00, 1.02)	**0.008**	1.09(1.07, 1.10)	**<0.001**	0.93(0.92, 0.95)	**<0.001**
**Living Situation**						
Live Alone	–		–		–	
Lives with non-using other(s)	0.57(0.54, 0.62)	**<0.001**	0.43(0.40, 0.47)	**<0.001**	0.97(0.87, 1.09)	0.59
Lives with other (s) who uses heroin or other opioids	1.79(1.68, 1.90)	**<0.001**	1.77(1.63, 1.92)	**<0.001**	1.57(1.42, 1.73)	**<0.001**
No stable arrangements / homeless	0.67(0.62, 0.73)	**<0.001**	0.68(0.62, 0.74)	**<0.001**	0.78(0.67, 0.91)	**0.001**
**Use any opioids AND a cocaine or amphetamine type stimulant at the same time in the past 30 days**						
0 days	–		–		–	
1–15 days	1.84(1.73, 1.96)	**<0.001**	1.83(1.69, 1.97)	**<0.001**	1.87(1.68, 2.09)	**<0.001**
16–30 days	1.41(1.31, 1.51)	**<0.001**	1.18(1.08, 1.29)	**<0.001**	2.15(1.92, 2.41)	**<0.001**
**Inject heroin in the past 30 days**						
0 days	–		–		–	
1–15 days	2.43(2.27, 2.60)	**<0.001**	3.48(3.21, 3.78)	**<0.001**	1.12(0.98, 1.28)	0.1
16–30 days	1.93(1.84, 2.02)	**<0.001**	1.66(1.55, 1.77)	**<0.001**	2.23(2.08, 2.40)	**<0.001**
**Use a fentanyl test strip**	1.08(1.08, 1.08)	**<0.001**	1.08(1.08, 1.09)	**<0.001**	1.06(1.06, 1.06)	**<0.001**
**Length of Opioid Use**	1(1.00, 1.01)	**<0.001**	1.02(1.02, 1.02)	**<0.001**	0.99(0.99, 1.0)	**<0.001**
**Opioid Withdraw in the last 30 days**						
None	–		–		–	
Moderate	0.43(0.41, 0.45)	**<0.001**	0.36(0.34, 0.39)	**<0.001**	0.54(0.50, 0.58)	**<0.001**
Severe	0.6(0.56, 0.64)	**<0.001**	0.41(0.37, 0.46)	**<0.001**	1(0.91, 1.10)	0.96
**Pain Severity Score**	0.94(0.94, 0.95)	**<0.001**	0.93(0.92, 0.94)	**<0.001**	0.98(0.97, 0.99)	**<0.001**
**Lifetime Overdose**						
No	–		–		–	
Yes	2.08(1.99, 2.17)	**<0.001**	2.51(2.36, 2.67)	**<0.001**	1.38(1.29, 1.47)	**<0.001**
**Lifetime Opioid Overdose**						
No	–		–		–	
** **Yes	2(1.91, 2.09)	**<0.001**	2.49(2.34, 2.64)	**<0.001**	1.29(1.21, 1.38)	**<0.001**
**Negative Life events**						
No	–		–		–	
Yes	1.03(0.98, 1.07)	0.24	0.96(0.90, 1.02)	0.14	1.22(1.14, 1.30)	**<0.001**
**Lifetime Arrest**						
Low	–		–		–	
Medium	0.72(0.68, 0.77)	**<0.001**	0.66(0.60, 0.71)	**<0.001**	1.09(1.01, 1.18)	**0.022**
High	1.07(1.01, 1.13)	**0.02**	1.23(1.14, 1.33)	**<0.001**	1.36(1.25, 1.48)	**<0.001**
**Lifetime Incarceration**						
0 days	–		–		–	
1–365 days	1.03(0.97, 1.09)	0.38	0.89(0.82, 0.98)	**0.015**	1.24(1.14, 1.35)	**<0.001**
More than a year	0.79(0.74, 0.84)	**<0.001**	0.78(0.72, 0.85)	**<0.001**	1.77(1.59, 1.97)	**<0.001**
**Days Using Heroin Alone**	0.96(0.95, 0.96)	**<0.001**	0.95(0.95, 0.96)	**<0.001**	0.98(0.97, 0.98)	**<0.001**

1 IRR = Incidence Rate Ratio, CI = Confidence Interval

Bold font indicates *p*<0.05.

**Table 3:** Longitudinal Multivariable Model of Protection with Naloxone Percentage on Full Sample and stratified by Gender (N = 422)

	Full Sample (N=422)	Male (N=268)	Female (N=150)
Characteristic	IRR^1,2^	IRR^1,2^	IRR^1,2^
**Visiting a SSP in the Last 3 Months**			
Never	–	–	–
Yes or Other Categories	1.94 (1.38, 2.74)***	1.84 (1.29, 2.64)***	0.72 (0.42, 1.23)
**Gender**			
Male	–		
Female	1.21 (0.82, 1.78)		
**Race**			
White	–	–	–
Black	0.73 (0.48, 1.12)	0.74 (0.43, 1.29)	0.93 (0.45, 1.92)
Latinx	0.64 (0.44, 0.94)*	0.69 (0.41, 1.15)	0.78 (0.42, 1.47)
Other	0.63 (0.34, 1.17)	0.66 (0.31, 1.41)	1.80 (0.46, 7.00)
**Age**			
20 to 40	–	–	–
41 to 50	0.67 (0.44, 1.01)	0.72 (0.43, 1.19)	0.52 (0.23, 1.17)
51 to 56	0.89 (0.53, 1.46)	1.60 (0.85, 3.01)	0.39 (0.16, 0.95)*
57 to 72	1.01 (0.56, 1.81)	1.54 (0.75, 3.17)	0.17 (0.05, 0.55)**
**Education**			
Did Not Complete High School	–	–	–
High School Graduate or GED	0.78 (0.55, 1.12)	0.46 (0.28, 0.73)**	1.48 (0.80, 2.73)
Some College or Greater	0.81 (0.56, 1.17)	0.47 (0.29, 0.76)**	2.31 (1.22, 4.36)**
**Social Support Score (Non User)**	0.89 (0.83, 0.95)***	0.97 (0.89, 1.05)	0.75 (0.66, 0.84)***
**Social Support Score (User)**	1.14 (1.07, 1.22)***	1.07 (0.98, 1.17)	1.30 (1.16, 1.46)***
**Living Situation**			
Live Alone	–	–	–
Lives with non-using other(s)	0.54 (0.37, 0.80)**	0.46 (0.29, 0.73)***	0.78 (0.34, 1.79)
Lives with other (s) who uses heroin or other opioids	0.93 (0.62, 1.40)	0.96 (0.59, 1.58)	0.57 (0.25, 1.29)
No stable arrangements / homeless	1.02 (0.66, 1.56)	0.77 (0.47, 1.27)	1.27 (0.51, 3.20)
**Use any opioids AND a cocaine or amphetamine type stimulant at the same time in the past 30 days**			
0 days	–	–	–
1–15 days	1.11 (1.08, 1.15)***	1.22 (1.17, 1.27)***	0.96 (0.92, 1.01)
16–30 days	1.32 (1.28, 1.37)***	1.22 (1.16, 1.28)***	1.36 (1.30, 1.42)***
**Inject heroin in the past 30 days**			
0 days	–	–	–
1–15 days	1.60 (1.49, 1.72)***	2.60 (2.33, 2.89)***	0.93 (0.84, 1.04)
16–30 days	0.92 (0.86, 0.98)*	0.92 (0.83, 1.02)	0.89 (0.80, 0.98)*
**Use a fentanyl test strip**	1.02 (1.02, 1.02)***	1.03 (1.02, 1.03)***	1.01 (1.01, 1.01)***
**Length of Opioid Use**	1.00 (0.98, 1.02)	1.00 (0.97, 1.02)	1.02 (0.98, 1.05)
**Opioid Withdraw in the last 30 days**			
None	–	–	–
Moderate	0.80 (0.78, 0.82)***	0.77 (0.74, 0.79)***	0.94 (0.90, 0.98)**
Severe	0.67 (0.64, 0.71)***	0.78 (0.72, 0.84)***	0.63 (0.59, 0.68)***
**Pain Severity Score**	0.96 (0.96, 0.96)***	0.96 (0.95, 0.96)***	0.96 (0.95, 0.97)***
**Lifetime Opioid Overdose**			
No	–	–	–
Yes	1.58 (1.17, 2.13)**	1.71 (1.19, 2.48)**	1.41 (0.81, 2.47)
**Negative Life events**			
No	–	–	–
Yes	0.77 (0.76, 0.79)***	0.86 (0.83, 0.88)***	0.71 (0.68, 0.74)***
**Lifetime Arrest**			
Low	–	–	–
Medium	1.90 (1.35, 2.66)***	2.28 (1.45, 3.60)***	1.03 (0.57, 1.84)
High	1.53 (1.08, 2.18)*	2.04 (1.29, 3.23)**	0.86 (0.46, 1.61)
**Days Using Heroin Alone**	0.99 (0.97, 1.00)*	0.99 (0.97, 1.00)	0.97 (0.95, 0.99)**

1 *p<0.05; **p<0.01; ***p<0.001

2 IRR = Incidence Rate Ratio

* controlled for visit and visit^2^ for the non-linear time effect

## Data Availability

At the conclusion of the study, all data will be made publicly available.
